# Potassium oxalurate monohydrate

**DOI:** 10.1107/S1600536809005637

**Published:** 2009-02-21

**Authors:** Lian-Feng Zhang

**Affiliations:** a College of Chemistry and Pharmacy Engineering, Nanyang Normal University, Nanyang 473061, People’s Republic of China

## Abstract

The title salt, poly[aqua-μ_3_-oxalurato-potassium(I)], [K(C_3_H_3_N_2_O_4_)(H_2_O)]_*n*_, which was obtained from a water solution of oxaluric acid and KOH at room temperature, crystallizes as potassium and oxalurate ions along with a water mol­ecule. The K^+^ cation lies on a crystallographic twofold rotation axis (site symmetry 2, Wyckoff position *f*), and the water and oxalurate mol­ecules are located within different mirror planes (site symmetry *m*, Wyckoff position *g*). The K^+^ cation is eight-coordinated by six O atoms of six oxalurate ligands and two O atoms from two water mol­ecules in a distorted square-anti­prismatic geometry. All of the eight coordinated O atoms are in a monodentate bridging mode, with alternate bridged K⋯K distances of 3.5575 (12) and 3.3738 (12) Å. The oxalurate ligand shows a μ_3_-bridging coordination mode, which links the K^+^ cation into a three-dimensional network. The oxalurate ligands and the water mol­ecules are involved in inter- and intra­molecular N—H⋯O, and O—H⋯O hydrogen bonds, which stabilize the network.

## Related literature

For oxalurate metal complexes, see: Falvello *et al.* (2002 ▶). For elongated K—O bonds, see: Karapetyan (2008 ▶); Kunz *et al.* (2009 ▶).
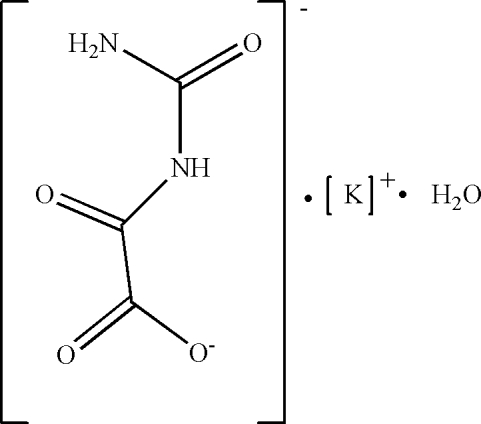


## Experimental

### 

#### Crystal data

[K(C_3_H_3_N_2_O_4_)(H_2_O)]
*M* *_r_* = 188.19
Orthorhombic, 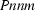

*a* = 7.7313 (17) Å
*b* = 12.799 (3) Å
*c* = 6.9313 (16) Å
*V* = 685.9 (3) Å^3^
*Z* = 4
Mo *K*α radiation
μ = 0.75 mm^−1^
*T* = 296 K
0.41 × 0.39 × 0.28 mm

#### Data collection

Bruker SMART CCD area-detector diffractometer
Absorption correction: multi-scan (*SADABS*; Bruker, 1997 ▶) *T*_min_ = 0.748, *T*_max_ = 0.816
3320 measured reflections
699 independent reflections
633 reflections with *I* > 2σ(*I*)
*R*_int_ = 0.013

#### Refinement

*R*[*F* ^2^ > 2σ(*F* ^2^)] = 0.026
*wR*(*F* ^2^) = 0.077
*S* = 1.09
699 reflections
66 parameters
H-atom parameters constrained
Δρ_max_ = 0.17 e Å^−3^
Δρ_min_ = −0.34 e Å^−3^

### 

Data collection: *SMART* (Bruker, 1997 ▶); cell refinement: *SAINT* (Bruker, 1997 ▶); data reduction: *SAINT*; program(s) used to solve structure: *SHELXS97* (Sheldrick, 2008 ▶); program(s) used to refine structure: *SHELXL97* (Sheldrick, 2008 ▶); molecular graphics: *SHELXTL* (Sheldrick, 2008 ▶); software used to prepare material for publication: *SHELXTL*.

## Supplementary Material

Crystal structure: contains datablocks I, global. DOI: 10.1107/S1600536809005637/si2155sup1.cif

Structure factors: contains datablocks I. DOI: 10.1107/S1600536809005637/si2155Isup2.hkl

Additional supplementary materials:  crystallographic information; 3D view; checkCIF report

## Figures and Tables

**Table 1:** Selected bond lengths (Å)

K1—O1	2.7291 (11)
K1—O3^i^	2.7812 (11)
K1—O5	2.8458 (13)
K1—O4^ii^	2.9775 (13)

Symmetry codes: (i) 
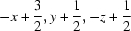
; (ii) 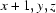.

**Table 2:** Hydrogen-bond geometry (Å, °)

*D*—H⋯*A*	*D*—H	H⋯*A*	*D*⋯*A*	*D*—H⋯*A*
N1—H1⋯O4^iii^	0.86	2.10	2.936 (2)	164
N2—H2*A*⋯O1^iv^	0.86	2.17	2.997 (2)	163
N2—H2*A*⋯O2^iv^	0.86	2.37	3.069 (2)	139
N2—H2*B*⋯O3	0.86	2.01	2.667 (2)	133
N2—H2*B*⋯O5^v^	0.86	2.38	3.076 (3)	138
O5—H1*W*⋯O2^i^	0.83	1.97	2.791 (2)	172
O5—H1*W*⋯O3^i^	0.83	2.59	3.068 (2)	118
O5—H2*W*⋯O2^vi^	0.83	2.14	2.973 (2)	178

Symmetry codes: (i) 
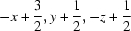
; (iii) 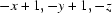; (iv) 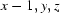; (v) 
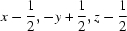
; (vi) 
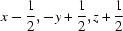
.

**Fig. 1.**
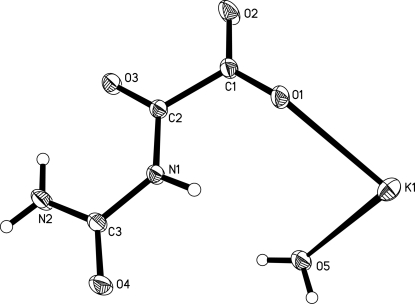
A perspective view of the asymmetric unit, showing the atomic numbering and displacement ellipsoids drawn at the 30% probability level.

**Fig. 2.**
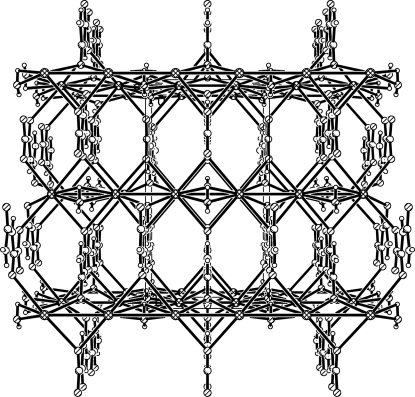
A view of the compound packing down the a axis.

## supplementary crystallographic information

### Comment

Oxaluric acid is the condensation product of oxalic acid and urea. Deprotonated oxalurate possesses four oxygen atoms and two amine N atoms, which can serve as hydrogen-bond acceptors and hydrogen-bond donors, respectively. In addition, one or more of six different atoms can bind to metal centers in any of at least three distinct coordination modes, namely, chelating, terminal, or bridging coordination (Falvello, 2002).

As shown in Fig. 1, the asymmetric structure unit consists of one K^+^ cation, one C_3_H_3_N_2_O_4_^-^ anion, and one water molecule. The K^+^ cation is surrounded by six oxalurate ligands and two water molecules, making close contacts with eight O atoms at 2.7291 (11)–2.9775 (13) A ° in a distorted square antiprismatic geometry of the central atom (Karapetyan, 2008; Kunz, 2009) (Table 1). All the eight coordinated O atoms are in the monodentate bridging mode, with the bridged K···K distance of 3.558 (1) and 3.374 (1) Å alternately. The oxalurate ligand, which is planar, shows a µ_3_-bridging coordination mode and links the K^+^ cation into a three-dimensional network (Fig. 2). The oxalurate ligands and water molecules are involved in inter- and intramolecular N—H···O, and O—H···O hydrogen bonds, which stabilize the network (Table 2).

### Experimental

A 10 ml sample of a KOH solution (0.5 mol/*L*) was added to a water suspension of oxaluric acid, HOOCCONHCONH_2_ (0.5 mmol/10 ml). The KOH addition produced a partial solubilization of the acid and then the precipitation of a white solid. After 20 min of stirring, the solid was filtered off, washed with i-PrOH. The single crystals suitable for X-ray analysis were obtained by slow diffusion of Et_2_O into the water solution of the solid.

### Refinement

Water H atoms were located in a difference Fourier and allowed to ride at the value approximately 0.83 Å with *U*_iso_(H) = 1.5*Ueq*(O). Other H atoms were positioned geometrically and treated as riding, with N—H = 0.86 Å (NH and NH_2_) and *U*_iso_(H) = 1.2*Ueq*(N).

### Crystal data

**Table:**

[K(C_3_H_3_N_2_O_4_)(H_2_O)]	*F*(000) = 384
*M**_r_* = 188.19	*D*_x_ = 1.823 Mg m^−^^3^
Orthorhombic, *P**n**n**m*	Mo *K*α radiation, λ = 0.71073 Å
Hall symbol: -P 2 2n	Cell parameters from 1939 reflections
*a* = 7.7313 (17) Å	θ = 2.9–28.2°
*b* = 12.799 (3) Å	µ = 0.75 mm^−^^1^
*c* = 6.9313 (16) Å	*T* = 296 K
*V* = 685.9 (3) Å^3^	Block, pink
*Z* = 4	0.41 × 0.39 × 0.28 mm

### Data collection

**Table:**

Bruker SMART CCD area-detector diffractometer	699 independent reflections
Radiation source: fine-focus sealed tube	633 reflections with *I* > 2σ(*I*)
graphite	*R*_int_ = 0.013
φ and ω scans	θ_max_ = 25.5°, θ_min_ = 3.1°
Absorption correction: multi-scan (*SADABS*; Bruker, 1997)	*h* = −9→9
*T*_min_ = 0.748, *T*_max_ = 0.816	*k* = −15→15
3320 measured reflections	*l* = −8→6

### Refinement

**Table:**

Refinement on *F*^2^	Primary atom site location: structure-invariant direct methods
Least-squares matrix: full	Secondary atom site location: difference Fourier map
*R*[*F*^2^ > 2σ(*F*^2^)] = 0.026	Hydrogen site location: inferred from neighbouring sites
*wR*(*F*^2^) = 0.077	H-atom parameters constrained
*S* = 1.09	*w* = 1/[σ^2^(*F*_o_^2^) + (0.0457*P*)^2^ + 0.2142*P*] where *P* = (*F*_o_^2^ + 2*F*_c_^2^)/3
699 reflections	(Δ/σ)_max_ < 0.001
66 parameters	Δρ_max_ = 0.17 e Å^−^^3^
0 restraints	Δρ_min_ = −0.34 e Å^−^^3^

### Fractional atomic coordinates and isotropic or equivalent isotropic displacement parameters (Å^2^)

**Table:**

	*x*	*y*	*z*	*U*_iso_*/*U*_eq_	
K1	1.0000	0.5000	0.24337 (6)	0.0336 (2)	
O1	0.89541 (18)	0.34475 (10)	0.0000	0.0329 (4)	
O2	0.90620 (19)	0.17010 (11)	0.0000	0.0478 (5)	
O3	0.55768 (18)	0.16337 (10)	0.0000	0.0349 (4)	
O4	0.30509 (18)	0.44721 (10)	0.0000	0.0396 (4)	
N1	0.5439 (2)	0.34235 (12)	0.0000	0.0278 (4)	
H1	0.6069	0.3977	0.0000	0.033*	
N2	0.2646 (2)	0.27328 (14)	0.0000	0.0414 (5)	
H2A	0.1538	0.2794	0.0000	0.050*	
H2B	0.3111	0.2122	0.0000	0.050*	
C1	0.8300 (3)	0.25554 (15)	0.0000	0.0266 (5)	
C2	0.6290 (2)	0.24914 (14)	0.0000	0.0239 (4)	
C3	0.3632 (2)	0.35754 (14)	0.0000	0.0281 (5)	
O5	0.7184 (2)	0.46547 (13)	0.5000	0.0490 (5)	
H1W	0.6919	0.5284	0.5000	0.073*	
H2W	0.6298	0.4287	0.5000	0.073*	

### Atomic displacement parameters (Å^2^)

**Table:**

	*U*^11^	*U*^22^	*U*^33^	*U*^12^	*U*^13^	*U*^23^
K1	0.0406 (3)	0.0241 (3)	0.0362 (4)	−0.00314 (16)	0.000	0.000
O1	0.0213 (7)	0.0206 (7)	0.0567 (10)	−0.0038 (5)	0.000	0.000
O2	0.0210 (7)	0.0224 (7)	0.0999 (15)	0.0037 (6)	0.000	0.000
O3	0.0226 (7)	0.0171 (7)	0.0650 (11)	−0.0027 (6)	0.000	0.000
O4	0.0227 (7)	0.0203 (7)	0.0758 (12)	0.0033 (6)	0.000	0.000
N1	0.0175 (8)	0.0165 (8)	0.0493 (11)	−0.0020 (6)	0.000	0.000
N2	0.0172 (8)	0.0219 (8)	0.0852 (16)	0.0006 (7)	0.000	0.000
C1	0.0194 (10)	0.0224 (9)	0.0379 (11)	−0.0015 (7)	0.000	0.000
C2	0.0197 (10)	0.0190 (9)	0.0330 (11)	−0.0007 (7)	0.000	0.000
C3	0.0184 (9)	0.0221 (9)	0.0438 (13)	0.0014 (7)	0.000	0.000
O5	0.0247 (8)	0.0271 (8)	0.0952 (14)	0.0024 (7)	0.000	0.000

### Geometric parameters (Å, °)

**Table:**

K1—O1	2.7291 (11)	O3—K1^vii^	2.7812 (11)
K1—O1^i^	2.7291 (11)	O3—K1^viii^	2.7812 (11)
K1—O3^ii^	2.7812 (11)	O4—C3	1.232 (2)
K1—O3^iii^	2.7812 (11)	O4—K1^v^	2.9775 (13)
K1—O5^iv^	2.8458 (13)	O4—K1^ix^	2.9775 (13)
K1—O5	2.8458 (13)	N1—C2	1.362 (2)
K1—O4^v^	2.9775 (13)	N1—C3	1.410 (2)
K1—O4^vi^	2.9775 (13)	N1—H1	0.8600
K1—K1^i^	3.3738 (12)	N2—C3	1.321 (3)
K1—K1^iv^	3.5575 (12)	N2—H2A	0.8600
K1—H1W	2.9950	N2—H2B	0.8600
O1—C1	1.249 (2)	C1—C2	1.556 (3)
O1—K1^i^	2.7291 (11)	O5—K1^iv^	2.8458 (13)
O2—C1	1.242 (2)	O5—H1W	0.8312
O3—C2	1.228 (2)	O5—H2W	0.8317
			
O1—K1—O1^i^	103.64 (4)	O3^ii^—K1—K1^iv^	50.24 (2)
O1—K1—O3^ii^	153.54 (4)	O3^iii^—K1—K1^iv^	50.24 (2)
O1^i^—K1—O3^ii^	84.00 (3)	O5^iv^—K1—K1^iv^	51.32 (2)
O1—K1—O3^iii^	84.00 (3)	O5—K1—K1^iv^	51.32 (2)
O1^i^—K1—O3^iii^	153.54 (4)	O4^v^—K1—K1^iv^	124.509 (19)
O3^ii^—K1—O3^iii^	100.48 (4)	O4^vi^—K1—K1^iv^	124.509 (19)
O1—K1—O5^iv^	136.56 (4)	K1^i^—K1—K1^iv^	180.0
O1^i^—K1—O5^iv^	92.67 (4)	C1—O1—K1	141.79 (2)
O3^ii^—K1—O5^iv^	66.81 (4)	C1—O1—K1^i^	141.79 (2)
O3^iii^—K1—O5^iv^	66.07 (4)	K1—O1—K1^i^	76.36 (4)
O1—K1—O5	92.67 (4)	C2—O3—K1^vii^	138.06 (4)
O1^i^—K1—O5	136.56 (4)	C2—O3—K1^viii^	138.06 (4)
O3^ii^—K1—O5	66.07 (4)	K1^vii^—O3—K1^viii^	79.52 (4)
O3^iii^—K1—O5	66.81 (4)	C3—O4—K1^v^	120.01 (9)
O5^iv^—K1—O5	102.63 (4)	C3—O4—K1^ix^	120.01 (9)
O1—K1—O4^v^	65.19 (4)	K1^v^—O4—K1^ix^	69.02 (4)
O1^i^—K1—O4^v^	73.70 (4)	C2—N1—C3	126.81 (16)
O3^ii^—K1—O4^v^	93.71 (3)	C2—N1—H1	116.6
O3^iii^—K1—O4^v^	131.30 (4)	C3—N1—H1	116.6
O5^iv^—K1—O4^v^	157.65 (5)	C3—N2—H2A	120.0
O5—K1—O4^v^	77.49 (3)	C3—N2—H2B	120.0
O1—K1—O4^vi^	73.70 (4)	H2A—N2—H2B	120.0
O1^i^—K1—O4^vi^	65.19 (4)	O2—C1—O1	127.80 (18)
O3^ii^—K1—O4^vi^	131.30 (4)	O2—C1—C2	115.29 (17)
O3^iii^—K1—O4^vi^	93.71 (3)	O1—C1—C2	116.91 (17)
O5^iv^—K1—O4^vi^	77.49 (3)	O3—C2—N1	124.44 (17)
O5—K1—O4^vi^	157.65 (5)	O3—C2—C1	119.70 (17)
O4^v^—K1—O4^vi^	110.98 (4)	N1—C2—C1	115.87 (16)
O1—K1—K1^i^	51.82 (2)	O4—C3—N2	123.36 (18)
O1^i^—K1—K1^i^	51.82 (2)	O4—C3—N1	119.31 (17)
O3^ii^—K1—K1^i^	129.76 (2)	N2—C3—N1	117.34 (17)
O3^iii^—K1—K1^i^	129.76 (2)	K1^iv^—O5—K1	77.37 (4)
O5^iv^—K1—K1^i^	128.68 (2)	K1^iv^—O5—H1W	92.2
O5—K1—K1^i^	128.68 (2)	K1—O5—H1W	92.2
O4^v^—K1—K1^i^	55.491 (19)	K1^iv^—O5—H2W	135.9
O4^vi^—K1—K1^i^	55.491 (19)	K1—O5—H2W	135.9
O1—K1—K1^iv^	128.18 (2)	H1W—O5—H2W	110.2
O1^i^—K1—K1^iv^	128.18 (2)		
			
O1^i^—K1—O1—C1	−177.2 (2)	K1^vii^—O3—C2—C1	−73.10 (14)
O3^ii^—K1—O1—C1	−72.9 (2)	K1^viii^—O3—C2—C1	73.10 (14)
O3^iii^—K1—O1—C1	28.56 (19)	C3—N1—C2—O3	0.0
O5^iv^—K1—O1—C1	73.8 (2)	C3—N1—C2—C1	180.0
O5—K1—O1—C1	−37.78 (19)	O2—C1—C2—O3	0.0
O4^v^—K1—O1—C1	−112.67 (19)	O1—C1—C2—O3	180.0
O4^vi^—K1—O1—C1	124.22 (19)	O2—C1—C2—N1	180.0
K1^i^—K1—O1—C1	−177.2 (2)	O1—C1—C2—N1	0.0
K1^iv^—K1—O1—C1	2.8 (2)	K1^v^—O4—C3—N2	−40.86 (6)
O1^i^—K1—O1—K1^i^	0.0	K1^ix^—O4—C3—N2	40.86 (6)
O3^ii^—K1—O1—K1^i^	104.25 (5)	K1^v^—O4—C3—N1	139.14 (6)
O3^iii^—K1—O1—K1^i^	−154.26 (4)	K1^ix^—O4—C3—N1	−139.14 (6)
O5^iv^—K1—O1—K1^i^	−109.03 (4)	C2—N1—C3—O4	180.0
O5—K1—O1—K1^i^	139.40 (3)	C2—N1—C3—N2	0.0
O4^v^—K1—O1—K1^i^	64.50 (3)	O1—K1—O5—K1^iv^	139.06 (3)
O4^vi^—K1—O1—K1^i^	−58.60 (3)	O1^i^—K1—O5—K1^iv^	−107.82 (4)
K1^iv^—K1—O1—K1^i^	180.0	O3^ii^—K1—O5—K1^iv^	−57.25 (3)
K1—O1—C1—O2	−92.22 (16)	O3^iii^—K1—O5—K1^iv^	56.75 (3)
K1^i^—O1—C1—O2	92.22 (16)	O5^iv^—K1—O5—K1^iv^	0.0
K1—O1—C1—C2	87.78 (16)	O4^v^—K1—O5—K1^iv^	−157.09 (5)
K1^i^—O1—C1—C2	−87.78 (16)	O4^vi^—K1—O5—K1^iv^	87.77 (8)
K1^vii^—O3—C2—N1	106.90 (14)	K1^i^—K1—O5—K1^iv^	180.0
K1^viii^—O3—C2—N1	−106.90 (14)		

Symmetry codes: (i) −*x*+2, −*y*+1, −*z*; (ii) −*x*+3/2, *y*+1/2, −*z*+1/2; (iii) *x*+1/2, −*y*+1/2, *z*+1/2; (iv) −*x*+2, −*y*+1, −*z*+1; (v) −*x*+1, −*y*+1, −*z*; (vi) *x*+1, *y*, *z*; (vii) *x*−1/2, −*y*+1/2, *z*−1/2; (viii) −*x*+3/2, *y*−1/2, −*z*+1/2; (ix) *x*−1, *y*, *z*.

### Hydrogen-bond geometry (Å, °)

**Table:**

*D*—H···*A*	*D*—H	H···*A*	*D*···*A*	*D*—H···*A*
N1—H1···O4^v^	0.86	2.10	2.936 (2)	164
N2—H2A···O1^ix^	0.86	2.17	2.997 (2)	163
N2—H2A···O2^ix^	0.86	2.37	3.069 (2)	139
N2—H2B···O3	0.86	2.01	2.667 (2)	133
N2—H2B···O5^vii^	0.86	2.38	3.076 (3)	138
O5—H1W···O2^ii^	0.83	1.97	2.791 (2)	172
O5—H1W···O3^ii^	0.83	2.59	3.068 (2)	118
O5—H2W···O2^x^	0.83	2.14	2.973 (2)	178

Symmetry codes: (v) −*x*+1, −*y*+1, −*z*; (ix) *x*−1, *y*, *z*; (vii) *x*−1/2, −*y*+1/2, *z*−1/2; (ii) −*x*+3/2, *y*+1/2, −*z*+1/2; (x) *x*−1/2, −*y*+1/2, *z*+1/2.
